# Bioprospecting of Brazilian Restinga Plants Reveals a *Myrciaria floribunda* Fraction with Apoptotic Activity Against Oral Carcinoma Cells

**DOI:** 10.3390/molecules31213976

**Published:** 2026-11-04

**Authors:** Julio Cesar Thurler-Júnior, Francisco Paiva Machado, Luiza Soares Batista, Diogo Folly Gomes Andrade, Gabriel Ouverney, Marcelo Guerra Santos, Anamel Blaudt Meira, Guilherme Freimann Wermelinger, Leandro Rocha, Bruno Kaufmann Robbs

**Affiliations:** 1 Laboratório de Rastreamento Biológico de Novas Drogas Contra o Câncer (CancerBioScreen), Departamento de Ciências Básicas, Instituto de Saúde de Nova Friburgo (ISNF), Universidade Federal Fluminense (UFF), Nova Friburgo CEP 28625-650, RJ, Brazil; juliothurler@id.uff.br (J.C.T.-J.); luizasb@id.uff.br (L.S.B.); ouverneymgabriel@gmail.com (G.O.); anamelmeira@gmail.com (A.B.M.); guilhermefwermelinger@gmail.com (G.F.W.); 2 Programa de Pós-Graduação em Ciências Aplicadas a Produtos para a Saúde, Faculdade de Farmácia, Universidade Federal Fluminense (UFF), Niterói CEP 24241-000, RJ, Brazil; fmachado@id.uff.br; 3 Laboratório de Tecnologia de Produtos Naturais (LTPN), Faculdade de Farmácia, Universidade Federal Fluminense (UFF), Niterói CEP 24241-000, RJ, Brazil; diogofolly@hotmail.com; 4 Programa de Pós-Graduação em Ciências Morfológicas, Instituto de Ciências Biomédicas, Universidade Federal do Rio de Janeiro, Rio de Janeiro CEP 21941-902, RJ, Brazil; 5 Departamento de Ciências da Faculdade de Formação de Professores (FFP), Universidade do Estado do Rio de Janeiro, Rio de Janeiro CEP 24435-005, RJ, Brazil; marceloguerrasantos@gmail.com

**Keywords:** oral squamous cell carcinoma, Myrtaceae, *Myrciaria floribunda*, bioprospecting, Atlantic Forest, apoptosis, selectivity index

## Abstract

Oral squamous cell carcinoma (OSCC) is the most prevalent malignancy of the head and neck region, and current chemotherapeutic regimens, based largely on platinum compounds, are limited by low selectivity and severe adverse effects. Aiming to identify selective cytotoxic agents from the Brazilian Atlantic Forest, we screened seventeen ethanolic extracts derived from ten plant species collected in Restinga ecosystems (*Myrciaria floribunda*, *Myrcia amazonica*, *Myrsine parvifolia*, *Myrcia ovata*, *Pilocarpus spicatus*, *Eugenia sulcata*, *Eugenia pruniformis*, *Ocotea indecora*, *Ocotea notata*, and *Ocotea pulchella*) against the OSCC cell line SCC-9 and primary human gingival fibroblasts. Among the active extracts, those from *M. floribunda* leaves and stem were the most potent and selective, with IC_50_ values of 78.7 and 56.9 µg/mL and selectivity indices of 7.14 and 6.80, respectively. Liquid–liquid partitioning of the stem extract yielded an ethyl acetate fraction (MfEA) that was further evaluated against a panel of eight tumor cell lines and against primary fibroblasts, displaying IC_50_ values ranging from 9.4 to 92.7 µg/mL and selectivity indices between 6.1 and 60.5. Chemical profiling of MfEA by UPLC-DAD-QTOF-ESI-HRMS/MS tentatively annotated gallic and ellagic acids, hydrolyzable tannins, and pentacyclic triterpenoids, several of which have individually been reported to exhibit antitumor activity. In SCC-9 cells, MfEA induced morphological hallmarks of apoptosis, activated effector caspases-3/7, increased the sub-G0/G1 population, and promoted phosphatidylserine externalization, without a detectable increase in H_2_O_2_ levels. MfEA exhibited negligible hemolytic activity (<3%) and, in C57BL/6 mice, an estimated intraperitoneal LD_50_ close to 200 mg/kg, with full tolerability at 100 mg/kg. Together, these findings identify the ethyl acetate fraction from *Myrciaria floribunda* as a promising lead from the Brazilian Restinga for further anticancer drug development.

## 1. Introduction

Head and neck cancer is among the most common malignancies worldwide, with approximately 890,000 new cases and 450,000 deaths reported in 2020, and oral squamous cell carcinoma (OSCC) accounts for up to 90% of all oral cancers [1,2]. Major risk factors for this malignancy include tobacco use, excessive alcohol consumption, and infection by oncogenic viruses such as Epstein–Barr virus and human papillomavirus (HPV) [3]. Treatment options for OSCC include surgery, chemotherapy, and radiotherapy, which may be used individually or in combination. Surgical intervention remains the primary therapeutic approach; however, clinical presentation and disease staging determine whether a single or multimodal treatment strategy will be employed [4]. Treatment planning is complex and often leads to negative impacts on patient quality of life, primarily due to the deleterious side effects of chemotherapeutic agents, which also entail high financial cost [5,6,7,8]. Moreover, mechanisms of resistance to antineoplastic agents have been increasingly elucidated, contributing to a worse prognosis of patients [9,10]. These challenges underscore the need for novel therapeutic alternatives for treating OSCC.

Natural products (NPs) remain a cornerstone of anticancer drug discovery. Among the 185 small-molecule anticancer drugs approved between 1981 and 2019, only 15.7% were of fully synthetic origin, while NPs and NP-derived compounds accounted for the majority of the remaining approvals [11,12]. Bioactive molecules derived from a wide variety of terrestrial and marine organisms have been shown to exhibit antidiabetic, antiparasitic, antiviral, antifungal, antimicrobial, antioxidant, anti-inflammatory, and anticancer activities [13,14,15,16]. Natural anticancer compounds exert their effects through multiple molecular mechanisms, including cell cycle arrest and induction of apoptosis, tumor growth inhibition via regulation of tumor suppressor genes, autophagy, or intracellular reactive oxygen species (ROS) levels, angiogenesis inhibition, and reversal of drug resistance in cancer cells [16]. Within the Plantae kingdom, species belonging to the Myrtaceae, Lauraceae, Primulaceae, and Rutaceae families have demonstrated various biological activities, such as antioxidant, cytotoxic, and anticancer effects [17,18], making them promising targets for anticancer research.

The Atlantic Forest is one of the largest biodiversity hotspots in the world [19]. Although it harbors more than 18,000 plant species, estimates of its remaining original cover range from 12% to 28%, depending on whether secondary and intermediate successional forests are included, making it a tropical domain highly vulnerable to climate change [20,21]. Within the Atlantic Forest, the Restinga is a sandy coastal ecosystem characterized by highly heterogeneous vegetation that faces extreme environmental conditions [22]. Biodiversity in these ecosystems is under significant threat from habitat loss and risk of disappearance, with projections indicating that climate change may drive woody plant species losses up to 19% on regional scales, highlighting the need for conservation and a deeper understanding of the chemical diversity of their secondary metabolites, which represents a largely untapped source of bioactive molecules [23,24]. Despite this potential, plants from the Restinga ecosystems remain poorly investigated as sources of anticancer agents, and, to the best of our knowledge, their selective cytotoxicity against OSCC has not been systematically evaluated.

Among the species that occur in Restinga ecosystems, *Myrciaria floribunda* (H. West ex Willd.) O. Berg (Myrtaceae), popularly known as “cambuí”, is a native tree of the Brazilian Atlantic Forest. Antioxidant, anti-inflammatory, and antinociceptive activities have been reported for this species [25,26,27] but its antitumor potential has been investigated in a single study, in which the ethyl acetate extract of the leaves inhibited the growth of a panel of human tumor cell lines [25]. Thus, the activity of *M. floribunda* against OSCC cells, the contribution of other plant organs, such as the stems, its selectivity toward normal cells, the mechanism underlying its cytotoxic effect, and its safety profile remain unknown.

To address these gaps, this study aimed to evaluate the cytotoxic and selective effects of seventeen ethanolic extracts from ten plant species collected in Restinga ecosystems against OSCC cells. Furthermore, the most promising extract was chemically partitioned, and the resulting fraction was chemically characterized and further investigated regarding its cytotoxicity, mechanism of cell death, selectivity profile, hemolytic potential, and acute toxicity in vivo.

## 2. Results and Discussion

### 2.1. Screening of Restinga Plants Reveals a Potent and Selective Extract

The extraction yields of the crude ethanolic extracts obtained from the investigated plant species are presented in Table 1. Among the species evaluated, *Myrciaria floribunda* yielded 21.74% and 8.24% for leaves and stems, respectively. Subsequent liquid–liquid partitioning of the leaf crude extract afforded hexane, dichloromethane, and ethyl acetate fractions with yields of 4.25%, 3.03%, and 3.63%, respectively. For the stem crude extract, the hexane, dichloromethane, and ethyl acetate fractions yielded 3.83%, 5.47%, and 3.27%, respectively.

The extraction yields varied considerably among the investigated species and plant organs (Table 1). In general, extracts from the leaves showed higher yields than stem extracts, indicating differences in the accumulation of extractable constituents between plant organs [28]. Additionally, the species evaluated belong to different botanical families and genera, mainly Myrtaceae and Lauraceae, which present distinct structural and chemical characteristics that can influence extraction efficiency. These factors, combined with the extraction capacity of ethanol, likely contributed to the observed variation in extract yields [29].

After plant material processing and extract production, the seventeen extracts derived from ten plant species were evaluated for their potential to reduce cancer cell viability in vitro. For this evaluation, we used the MTT assay, an indirect measurement of cell viability based on the conversion of 3-(4,5-dimethylthiazol-2-yl)-2,5-diphenyltetrazolium bromide into formazan by viable cells [30]. Carboplatin, a platinum-based chemotherapeutic agent widely used in OSCC therapy, served as the positive control.

The extracts were initially screened in SCC-9 cells. The half-maximal inhibitory concentration (IC_50_) could be calculated for nine extracts, namely the leaf and stem extracts of *Myrciaria floribunda*, the leaf extracts of *Myrcia ovata*, *Pilocarpus spicatus*, and *Eugenia sulcata*, the leaf and stem extracts of *Eugenia pruniformis*, and the leaf and stem extracts of *Ocotea indecora* (Table 2).

The leaf and stem extracts of *Myrcia amazonica*, *Myrsine parvifolia*, and *Ocotea notata*, as well as the stem extract of *Myrcia ovata* and the leaf extract of *Ocotea pulchella*, did not exhibit significant cytotoxicity within the tested concentration range (Table 2).

The active extracts displayed IC_50_ values ranging from 56.90 to 169.0 μg/mL (Table 2). Only the stem and leaf extracts of *M. floribunda* were more potent than carboplatin (IC_50_ = 97.70 μg/mL), by 1.7- and 1.2-fold, respectively, whereas the remaining seven active extracts showed IC_50_ values between 106.4 and 169.0 μg/mL, up to 1.7-fold higher than that of the reference drug.

In addition to potency, selectivity is a critical attribute for anticancer drug candidates, defined as the ratio between cytotoxicity in normal cells and cytotoxicity in tumor cells. Evaluating selectivity is a key step in early drug discovery, as it anticipates the risk of off-target toxicity in normal tissues and underlies the therapeutic window of the drug candidate [31]. To assess this parameter, we tested seven of the nine active extracts in primary human gingival fibroblasts. The same primary human gingival fibroblasts were used as the non-tumor reference for all cell lines, so that all SI values were obtained under identical experimental conditions; for the non-oral and murine tumor lines, the SI therefore expresses cytotoxicity relative to this common reference rather than tissue- or species-matched selectivity.

After obtaining the IC_50_ values in normal cells, we calculated the selectivity index (SI), defined as the ratio of the IC_50_ in normal cells to the IC_50_ in cancer cells (Table 2). Although selectivity thresholds vary across studies, SI values above 2 are commonly adopted to indicate preferential activity toward tumor cells [32]. According to this classification, only the stem and leaf extracts of *Myrciaria floribunda* and the stem extract of *Eugenia pruniformis* and *Ocotea indecora* were considered selective (Table 2). In addition, carboplatin was below the selectivity threshold, consistent with the well-documented toxic effects of platinum-based chemotherapy in both experimental and clinical settings [33,34,35].

*Myrciaria floribunda* (H. West ex Willd) is a tree species belonging to the Myrtaceae family, native to the Brazilian Atlantic Forest, where it is popularly known as ‘cambuí’ or ‘camboim amarelo’. Previous reports have described biological activities for *M. floribunda*, including antioxidant activity for fruit peels and leaves [25] and anti-inflammatory effects [26]. However, to the best of our knowledge, the cytotoxic and antitumor potential of *M. floribunda* extracts against OSCC cells has not been previously reported.

Taken together, these results show that *M. floribunda* extracts were more potent and selective than carboplatin, the standard treatment for OSCC. Accordingly, the crude stem and leaf ethanolic extracts were submitted to liquid–liquid partitioning with hexane, dichloromethane, and ethyl acetate, and the resulting fractions were evaluated in three OSCC cell lines (SCC-4, SCC-9, and SCC-25) and in primary gingival fibroblasts.

### 2.2. The Ethyl Acetate Fraction of Myrciaria floribunda Stem Is Potent and Highly Selective

Liquid–liquid partitioning of the crude stem and leaf extracts was performed sequentially with hexane, dichloromethane, and ethyl acetate, and each of the resulting partitions was re-evaluated against three OSCC cell lines (SCC-4, SCC-9, and SCC-25) and primary human gingival fibroblasts using the MTT assay (Table 3).

Overall, the *M. floribunda* leaf fractions showed limited selectivity despite appreciable potency. The ethyl acetate fraction showed no activity against any of the SCC cell lines. The dichloromethane fraction was active only against SCC-9 cells but showed no selectivity (SI 1.6). In contrast, the hexane fraction exhibited substantial cytotoxicity against all SCC cell lines (IC_50_: 19.6–38.1 µg/mL) but was selective only against SCC-9 (SI: 3.49).

Interestingly, although liquid–liquid partitioning increased the cytotoxic potency of the nonpolar fractions, a reduction in selectivity toward tumor cells was observed. This finding may suggest that the crude extract contains compounds that modulate the biological activity of the cytotoxic constituents, possibly through synergistic interactions that contribute to tumor selectivity. Considering this scenario, the leaf-derived fractions were not further investigated. Carboplatin was less potent than the hexane partition in all three SCC cell lines and exceeded the selectivity threshold only in SCC-4.

Extracts from *M. floribunda* leaves have previously been reported to exhibit cytotoxic activity against cancer cells, although the selectivity index was not evaluated [25]. In agreement with this report, the leaf-derived fractions evaluated in the present study displayed moderate cytotoxicity and limited selectivity.

In contrast, the fractions of *M. floribunda* obtained from the stems were cytotoxic against all SCC cell lines. The hexane fraction showed potent cytotoxicity against SCC-9 cells; however, it was less selective overall. The dichloromethane fraction was the most potent of the three, exhibiting strong activity against all SCC cell lines (IC_50_: 14.4–23.1 µg/mL), but with more modest selectivity (SI: 2.81–4.52). The ethyl acetate fraction was the least potent (42.9–71.3 µg/mL), but it showed remarkable selectivity, with SI values above 7 across all SCC cell lines and a maximum SI of 13.2 against SCC-9 cells. Based on this superior selectivity profile, the ethyl acetate fraction of the *M. floribunda* stem extract (MfEA) was selected for further assessment of its putative chemical profile by UPLC-DAD-QTOF-ESI-HRMS/MS and for evaluation of its cytotoxicity in cancer cell lines from other tissues.

**Figure 1:**
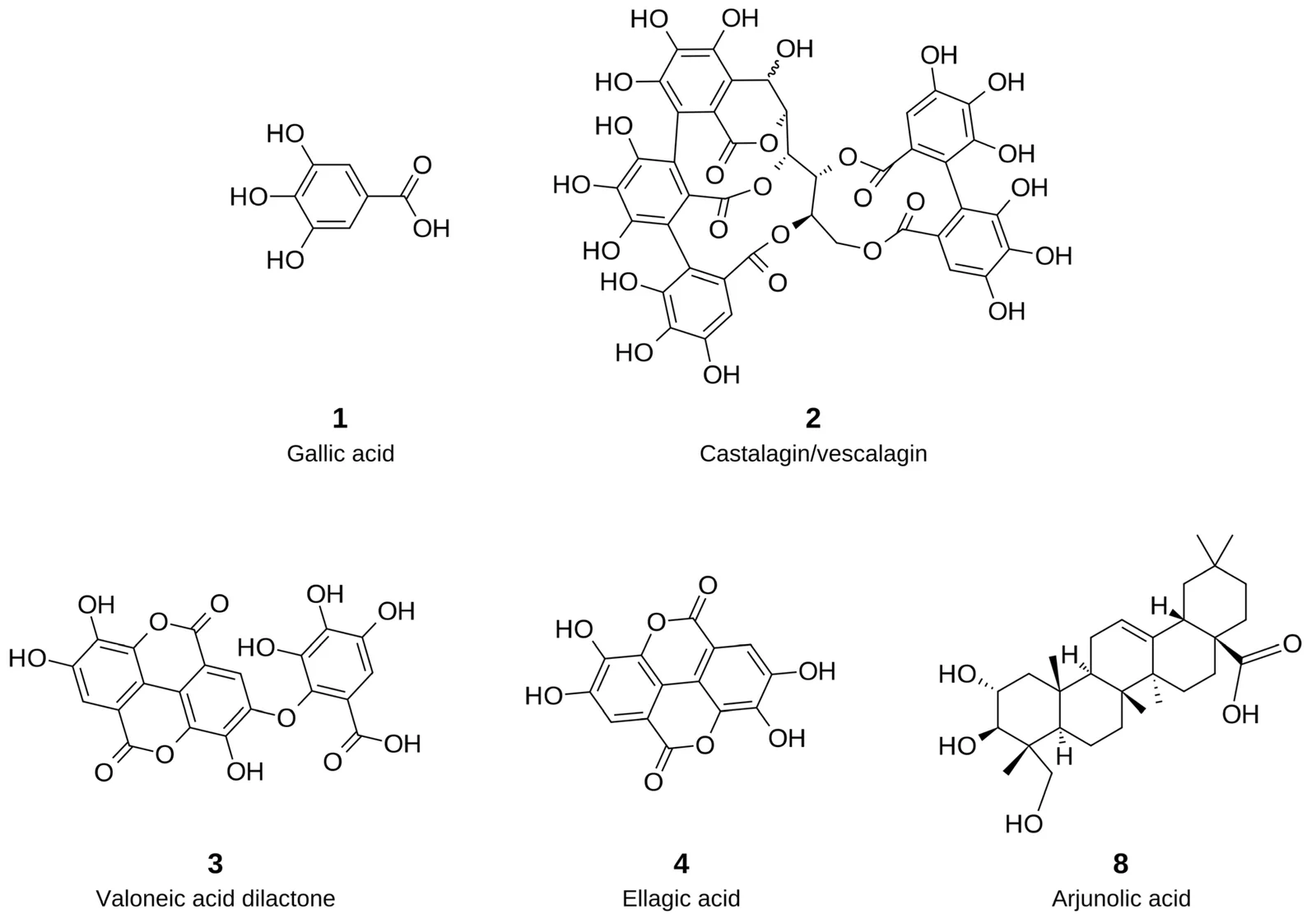
Chemical structures of the main metabolites putatively annotated in the ethyl acetate fraction of *Myrciaria floribunda* stems (MfEA). Compounds are numbered according to their elution order in Table 4: gallic acid (**1**), castalagin/vescalagin (**2**), valoneic acid dilactone (**3**), ellagic acid (**4**), and arjunolic acid (**8**). Structures were proposed on the basis of accurate mass, molecular formula, MS/MS fragmentation, and comparison with literature data, without confirmation by authentic standards, and therefore correspond to putatively annotated compounds (level 2 of the Metabolomics Standards Initiative) [57]. The wavy bond in **2** indicates that the configuration at C-1 was not assigned, because castalagin and vescalagin are C-1 epimers that cannot be distinguished by MS/MS. Stereochemistry is depicted as reported for the known compounds and was not determined in the present study. The O-methylated ellagic acid derivatives and the ursane/oleanane- and lupane-type triterpenoids listed in Table 4 are not depicted, because the position of the methoxy groups and the triterpene skeleton could not be established.

The chromatographic analysis allowed the putative identification of several polyphenolic compounds, including gallic acid (**1**), castalagin/vescalagin isomers (**2**), valoneic acid dilactone (**3**), ellagic acid (**4**), and O-methylated ellagic acid derivatives (**5**–**7**) (Table 4 and Figure 1). The chemical composition putatively annotated is consistent with the intermediate polarity of ethyl acetate, which preferentially partitions phenolic compounds of moderate polarity owing to its intermediate dielectric constant and hydrogen-bond-accepting properties. In addition to these phenolics, a smaller number of pentacyclic triterpenoids, including arjunolic acid (**8**) and ursane/oleanane- and lupane-type triterpenes (**9**, **10**), were also putatively observed.

The putative phytochemical profile of the ethyl acetate fraction is consistent with previous reports for the genus *Myrciaria*, in which phenolic compounds represent the predominant class of secondary metabolites, particularly tannins, phenolic acids, and flavonoids [36]. Gallic acid (**1**) and ellagic acid (**4**) have previously been reported in *M. floribunda fruits*, *M. dubia* fruits and seeds, and *M. glazioviana* fruits, whereas hydrolyzable tannins and ellagitannins have also been described in *Myrciaria* species [27,37,38,39,40]. Likewise, pentacyclic triterpenoids have also been reported in species of the genus *Myrciaria*, further supporting their occurrence as characteristic constituents of this genus [39].

**Table 4:** Putative annotated metabolites in the ethyl acetate fraction of *Myrciaria floribunda* stems by UPLC-DAD-QTOF-ESI-HRMS/MS in negative and positive ionization modes. RT = retention time. *m/z* = observed mass-to-charge ratio of the deprotonated [M–H]^−^ or protonated [M+H]^+^ ion. Formula = molecular formula of the neutral compound. Error = mass error between observed and calculated *m/z*. No. = compound number, assigned by elution order; structures of **1**–**4** and **8** are depicted in Figure 1.

No.	Annotation	RT (min)	*m/z*	Formula	MS/MS Fragments (*m/z*)	Error (ppm)	Ref.
**Negative ionization mode, [M–H]^−^**							
**1**	Gallic acid	8.2	169.0133	C_7_H_6_O_5_	125.0237	5.6	[41]
**2**	Castalagin/vescalagin isomer	9.8	933.0702	C_41_H_26_O_26_	631.0595, 300.9973, 275.0180, 249.0401	6.7	[42]
**3**	Valoneic acid dilactone	10.1	469.0047	C_21_H_10_O_13_	407.0036, 379.0130, 299.9885	1.5	[43]
**4**	Ellagic acid	16.3	300.0010	C_14_H_6_O_8_	200.0110, 145.0296	1.9	[44]
**5**	*O*-Methylellagic acid isomer I	20.0	315.0147	C_15_H_8_O_8_	299.9905, 270.9859, 216.0038	0.3	[45]
**6**	*O*-Methylellagic acid isomer II	20.9	315.0159	C_15_H_8_O_8_	248.9676, 198.9218, 146.9668	3.9	[45]
**7**	Di-*O*-methylellagic acid isomer	28.4	329.0304	C_16_H_10_O_8_	285.0054, 270.9881, 242.9930, 215.0003	0.3	[46]
**8**	Arjunolic acid	35.8	487.3450	C_30_H_48_O_5_	248.9721	3.1	[47]
**9**	Ursane/oleanane-type triterpenic acid	36.6	485.3302	C_30_H_46_O_5_	248.9712, 198.9224	6.2	[48]
**Positive ionization mode, [M+H]^+^**							
**1**	Gallic acid	8.2	171.0290	C_7_H_6_O_5_	127.0385, 109.9585	1.0	[49]
**2**	Castalagin/vescalagin isomer	9.7	935.0830	C_41_H_26_O_26_	615.0579, 495.0223, 469.0415	4.8	[50]
**4**	Ellagic acid	16.2	303.0135	C_14_H_6_O_8_	201.0193, 145.0293	7.6	[51]
**5**	*O*-Methylellagic acid isomer	19.9	317.0303	C_15_H_8_O_8_	257.0100, 200.0115	3.6	[52]
**7**	Di-*O*-methylellagic acid isomer	28.2	331.0467	C_16_H_10_O_8_	221.0826	5.6	[53]
**10**	Lupane triterpenoid	35.8	453.3383	C_30_H_44_O_3_	255.2111, 215.1800, 201.1647, 119.0859	4.4	[48]

To the best of our knowledge, this is the first report describing the putative chemical profile of the ethyl acetate fraction obtained from *M. floribunda* stems. Previous phytochemical studies on this species have primarily focused on fruits, leaves, fruit peel, and essential oils, whereas information regarding stem extracts remains underexplored [25,54,55,56]. Although UPLC-DAD-QTOF-ESI-HRMS/MS analysis provided valuable insights into the possible chemical composition of the ethyl acetate fraction, the detected metabolites were tentatively annotated based on their mass spectrometric characteristics and literature data. Therefore, their structural identities remain to be confirmed using complementary analytical approaches and authentic standards. Furthermore, the constituents underlying the observed cytotoxic activity have not yet been established. In addition, because MfEA was prepared as a single batch, its batch-to-batch consistency has not yet been established. Future studies involving quantitative chemical profiling and bioactivity-guided fractionation, followed by isolation and structural characterization, are needed to identify the active constituents and clarify their contribution to the biological effects.

Among the metabolites putatively annotated in MfEA, five could be assigned to single, defined chemical structures, which are shown in Figure 1: gallic acid (**1**), castalagin/vescalagin (**2**), valoneic acid dilactone (**3**), ellagic acid (**4**), and arjunolic acid (**8**). These compounds represent the most reliable putative annotations of the fraction and include the constituents to which the antitumor activities discussed below have been attributed in the literature. The O-methylated ellagic acid derivatives and the remaining triterpenoids were not depicted, because the position of the methoxy groups and the triterpene skeleton, respectively, could not be assigned from the available MS/MS data.

In addition to the SCC cell lines, MfEA cytotoxicity was evaluated against five additional cancer cell lines: murine melanoma (B16-F10), human colon carcinoma (HCT 116), human cervical carcinoma (HeLa), human hepatocellular carcinoma (HepG2), and murine mammary carcinoma (4T1) (Table 5). MfEA showed IC_50_ values of the same order of magnitude across the eight cancer cell lines, except for 4T1, where it exhibited markedly higher cytotoxicity (9.4 µg/mL). The fraction was selective for all cancer cell lines, with particularly high selectivity against 4T1 cells, achieving a selectivity index of 60.5.

Because 4T1 and B16-F10 are murine cell lines, whereas the reference fibroblasts are human, the SI values obtained for these two lines involve an interspecies comparison and should be interpreted with caution.

MfEA was more potent than carboplatin in six of the eight cell lines tested. Regarding selectivity, carboplatin was selective only for 4T1, HepG2, HeLa, and SCC-4 cells, and even against those cell lines it showed lower SI values than MfEA. Taken together, these results indicate that MfEA was, on average, both more potent and markedly more selective than the standard chemotherapeutic agent carboplatin.

It should be noted, however, that MfEA is a complex mixture of phytochemicals, whereas carboplatin is a defined drug already used in clinical practice, and the two differ substantially in composition and molecular characteristics. Because these comparisons were based on mass concentrations, they reflect the relative cytotoxicity of MfEA and carboplatin under the in vitro conditions tested and do not establish the therapeutic superiority of the fraction.

A previous study reported the antiproliferative activity of an ethyl acetate fraction obtained from *M. floribunda* leaves against a panel of tumor cell lines; however, that evaluation was restricted to growth inhibition assays [25]. The present study extends these findings in several respects: it shows that the antitumor constituents may also be present in the stems, establishes the selectivity of MfEA across eight tumor cell lines using primary human gingival fibroblasts as a normal reference, and, as described in the following sections, characterizes its mechanism of cell death and evaluates its hemolytic potential and acute toxicity in vivo.

Several of the metabolites putatively annotated in MfEA, including gallic acid (**1**), ellagic acid (**4**), hydrolyzable tannins, and pentacyclic triterpenoids, have individually been associated with antitumor activity [58,59,60,61]. Gallic acid showed IC_50_ values of 28.5 and 22.1 µg/mL against the hepatocellular carcinoma cell lines HepG2 and SMMC-7721, respectively [62], and was more cytotoxic to the squamous cell carcinoma cell line A431 (IC_50_ = 7.15 µg/mL) than to non-tumorigenic HaCaT keratinocytes (IC_50_ = 13.29 µg/mL) [63]. Ellagic acid displayed selective cytotoxicity against the OSCC cell lines CAL-27 and SCC-9, with minimal toxicity toward keratinocytes [64]. Ellagitannins inhibit tumor cell proliferation and induce apoptosis [60,65], and lupane-, oleanane-, and ursane-type triterpenoids exert cytotoxic and antiproliferative effects against a broad range of tumor cell lines [61,66]. These constituents are therefore likely to contribute to the activity of MfEA. However, the biological activity of plant fractions generally reflects the combined action of multiple constituents, and it would therefore be premature to attribute the effects of MfEA to any single compound. The activity observed here is more likely to result from the collective contribution of the putative metabolites described in the fraction.

In contrast, evidence of antitumor activity for valoneic acid dilactone (**3**) and arjunolic acid (**8**) remains scarce. Valoneic acid dilactone has been characterized mainly as a potent inhibitor of the endoribonuclease RNase L, and it was not cytotoxic to A549 lung adenocarcinoma cells at concentrations up to 25 µM [67]. Arjunolic acid has been used mainly as a scaffold for semisynthetic amide derivatives, which displayed activity against the NCI-60 tumor cell panel superior to that of the parent triterpenoid [68]. To our knowledge, neither compound has been evaluated against OSCC cells, and their contribution to the activity of MfEA, if any, remains to be established.

Taken together, the putative chemical profile of MfEA revealed a fraction enriched in phenolic acids, hydrolyzable tannins, and pentacyclic triterpenoids, metabolite classes consistently associated with antitumor activity. Since several of these constituents have been reported to induce apoptosis in tumor cells, the mechanisms underlying the cytotoxic effect of MfEA against SCC-9 cells were investigated.

### 2.3. MfEA Induces Caspase-3/7 Activation and Apoptosis

**Figure 2:**
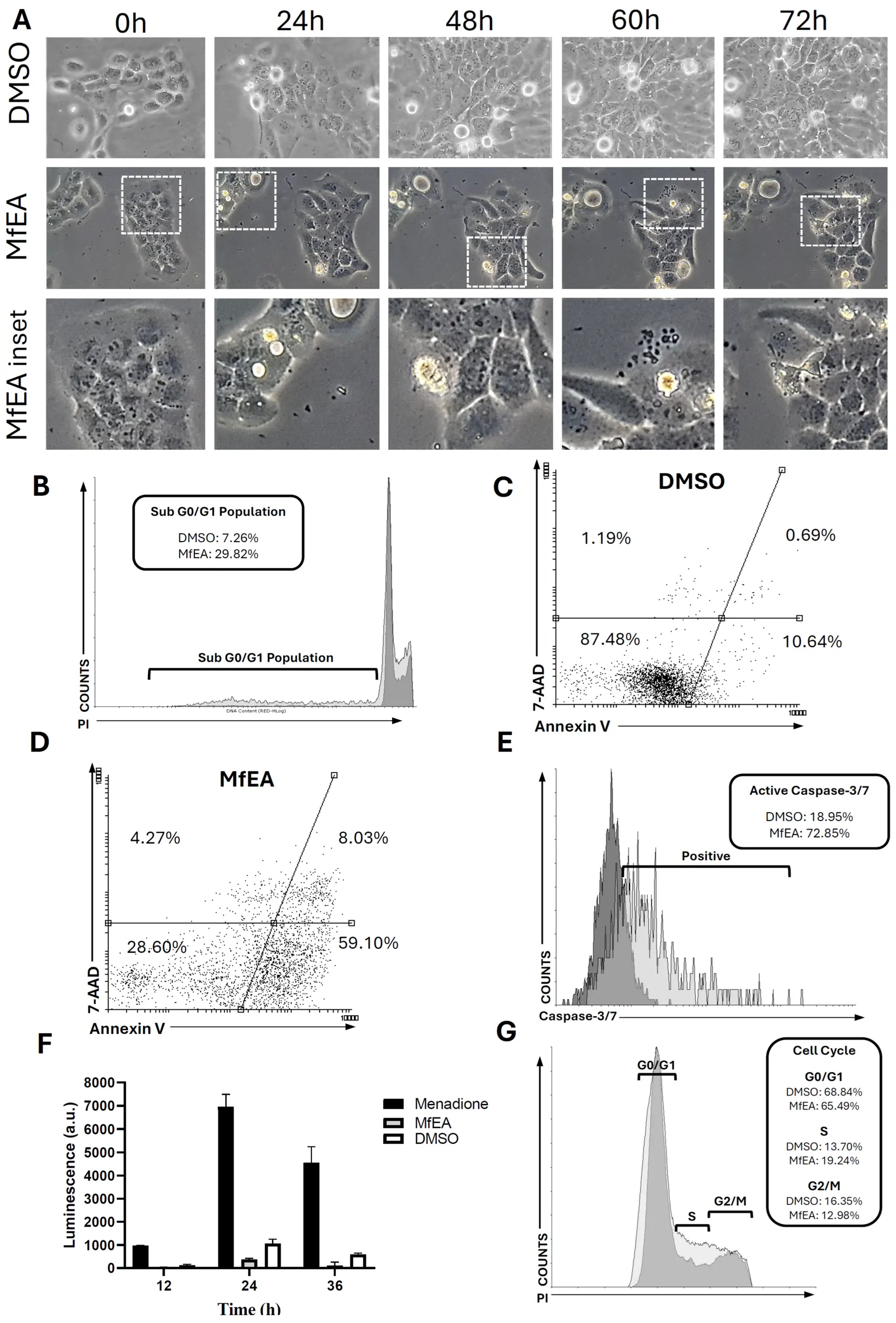
MfEA induces apoptotic features in SCC-9 cells. (**A**) Representative time-lapse videomicroscopy images of SCC-9 cells treated with MfEA (2 × IC_50_) or DMSO (negative control) for 72 h, showing morphological features consistent with apoptosis. White dashed boxes indicate the areas shown at 10× magnification in the insets. (**B**) Representative histogram highlighting the sub-G0/G1 population in SCC-9 cells following treatment with MfEA (2 × IC_50_) or DMSO for 48 h. (**C**,**D**) Representative Annexin V/7-AAD dot plots of SCC-9 cells treated with DMSO (**C**) or MfEA (**D**) for 48 h, showing the apoptotic cell population exposing phosphatidylserine. (**E**) Representative histogram of caspase-3/7 activation assessed by flow cytometry in SCC-9 cells treated with MfEA (2 × IC_50_) or DMSO for 48 h. (**F**) Hydrogen peroxide (H_2_O_2_) levels measured by luminescence using the ROS-Glo H_2_O_2_ assay in SCC-9 cells treated with MfEA (2 × IC_50_), DMSO (negative control), or menadione (20 μM, positive control) for 12, 24 and 36 h. (**G**) Representative cell cycle distribution of SCC-9 cells stained with propidium iodide and analyzed by flow cytometry after 48 h of treatment with MfEA (2 × IC_50_) or DMSO. In (**B**), (**E**), and (**G**), dark grey and light grey shaded areas correspond to DMSO- and MfEA-treated cells, respectively.

To generate hypotheses about the cell death pathway induced by MfEA, SCC-9 cells were treated with MfEA at 2 × IC_50_ for 72 h, and morphological changes were monitored by time-lapse videomicroscopy. MfEA treatment induced cell shrinking, detachment, and membrane blebbing, all of which are morphological hallmarks of apoptosis [69,70]. In contrast, cells treated with the negative control (DMSO) displayed normal proliferation throughout the imaging period (Figure 2A).

To further investigate the mechanisms underlying MfEA-induced cell death, we evaluated DNA fragmentation by analyzing the sub-G0/G1 population using propidium iodide staining and flow cytometry. SCC-9 cells treated with MfEA for 48 h exhibited a marked increase in the sub-G0/G1 population (29.8%) compared to the DMSO-treated group (7.2%) (Figure 2B). The sub-G0/G1 population is widely accepted as representing cells with fragmented DNA, a characteristic feature of apoptosis [71]. These findings suggest that MfEA induces apoptotic cell death in SCC-9 cells.

To independently confirm activation of the apoptotic pathway, phosphatidylserine externalization, a well-established early hallmark of apoptosis, was assessed by flow cytometry using Annexin V/7-AAD staining [72]. The DMSO-treated group showed a high percentage of viable cells (87.5%) and only 10.6% of apoptotic cells (Figure 2C). In contrast, MfEA-treated cells exhibited a markedly reduced percentage of viable cells (28.6%) and an increased percentage of apoptotic cells (59.0%), representing more than a threefold increase compared to the control (Figure 2D). Together, these findings further support MfEA-induced apoptosis in SCC-9 cells.

Based on morphological and biochemical evidence of apoptosis, we subsequently evaluated effector caspase-3/7 activation by flow cytometry using a fluorogenic substrate. SCC-9 cells treated with MfEA exhibited 72.8% of cells with active caspase-3/7, whereas only 18.9% of cells in the DMSO-treated group showed activation, representing an approximately four-fold increase (Figure 2E). Caspase-3/7 activation is a central event in the execution phase of apoptosis, leading to the cleavage of multiple intracellular substrates involved in chromatin condensation, membrane blebbing, apoptotic body formation, and DNA fragmentation [73]. Together with the apoptotic morphology and the increase in the sub-G0/G1 population described above, these findings are consistent with MfEA causing cell death through the apoptotic pathway.

This apoptotic profile is in line with the activity described for the metabolite classes identified in MfEA. Ellagic acid (**4**), one of the compounds identified in MfEA, induces caspase-3 activation in tumor cells, and its apoptotic effect is abolished by the pan-caspase inhibitor z-VAD-fmk, confirming a caspase-dependent mechanism [74]. Consistent findings have been reported in other tumor models, in which ellagic acid inhibited cell growth and triggered apoptosis through a caspase-dependent pathway [75].

Similarly, plant extracts containing castalagin/vescalagin-type ellagitannins (**2**) together with ellagic acid reduce cell viability and induce apoptosis in a panel of human tumor cell lines [76], and hydrolyzable tannins structurally related to those detected in MfEA promote apoptotic cell death in human oral tumor cell lines, characterized by DNA fragmentation and caspase-mediated substrate cleavage [77]. Ellagitannins and their derivatives also induce phosphatidylserine externalization, caspase-3 activation, and PARP cleavage in colon carcinoma cells [78]. Gallic acid (**1**), likewise, identified in MfEA, reduces viability and proliferation of cervical carcinoma cells while sparing normal cells [79], acting through both caspase-dependent and caspase-independent pathways [80].

Pentacyclic triterpenoids, also putative annotated in MfEA, converge on the same execution pathway. Lupane-, oleanane-, and ursane-type triterpenoids induce selective apoptosis in tumor cells through regulation of BCL-2 and BH3 family proteins [81], and ursane- and oleanane-type triterpenic acids activate caspase-3/7 and caspase-9 in leukemia cells [82]. Taken together, the apoptotic response elicited by MfEA is compatible with the combined contribution of phenolic acids, hydrolyzable tannins, and triterpenoids annotated in the fraction, whose reported activities converge on caspase-3/7 activation and DNA fragmentation.

Reactive oxygen species (ROS) play a central role in the modulation of apoptotic pathways triggered by many anticancer agents, including several natural products [83,84]. Because oxidative stress has been implicated in the cytotoxicity of some phenolic constituents tentatively annotated in MfEA, we investigated whether MfEA-induced apoptosis was associated with changes in hydrogen peroxide (H_2_O_2_) levels. Under the conditions tested (2 × IC_50_ for 12, 24 and 36 h), MfEA did not increase H_2_O_2_ levels relative to the DMSO control (Figure 2F), whereas the positive control menadione, a known redox-cycling agent, induced a robust increase in H_2_O_2_.

The absence of detectable H_2_O_2_ accumulation in MfEA-treated cells contrasts with the mechanism most frequently reported for isolated phenolic compounds, which are often described as pro-oxidant in tumor cells. However, apoptosis without detectable ROS accumulation has been documented for compounds structurally related to those putatively annotated in MfEA. Gallic acid (**1**), for instance, has been shown to induce HeLa cell death primarily through glutathione depletion rather than through increased ROS levels [85]. In human oral tumor cell lines, the apoptosis-inducing activity of hydrolyzable tannins was not abolished by catalase, arguing against a central role for hydrogen peroxide in their cytotoxic effect [77]. Likewise, the lupane-type triterpenoid betulinic acid induced caspase-dependent apoptosis in bladder carcinoma cells without altering intracellular ROS levels [86].

In addition, the pro-oxidant behavior of polyphenols is markedly concentration- and context-dependent, and dual antioxidant/pro-oxidant activities have been reported for both gallic (**1**) and ellagic (**4**) acids [87]. In complex matrices such as MfEA, the antioxidant capacity of the phenolic pool may therefore offset the oxidative burden generated by individual constituents. These observations are consistent with an apoptotic effect of MfEA that occurs without detectable H_2_O_2_ accumulation under the conditions evaluated, similar to the behavior described for hydrolysable tannins and lupane triterpenoids in tumor cells.

To further characterize the mechanism underlying MfEA-induced cell death, cell cycle distribution was analyzed by propidium iodide staining and flow cytometry. MfEA induced a slight increase in the S-phase population (19.2% vs. 13.7% in the DMSO-treated group), accompanied by minor reductions in G1 (65.5% vs. 68.8%) and G2/M (13.0% vs. 16.4%); however, no cell cycle arrest was observed (Figure 2G). These findings suggest that MfEA induces apoptosis without a preceding cell cycle arrest.

The absence of a marked cell cycle arrest distinguishes MfEA from the profile reported for some of its individual constituents tentatively annotated. Ellagic acid (**4**) has been shown to induce a slight G1 arrest in colon carcinoma cells, whereas its gut-derived metabolites promote arrest at the G2/M checkpoint [78], and urolithins have been reported to arrest cell cycle progression at the S phase in colon carcinoma cells [88]. Ellagic acid has similarly been described to induce G0/G1 arrest in bladder carcinoma cells through p53/p21 upregulation [89]. The modest accumulation in S phase observed for MfEA is compatible with these reports, although in the present study apoptosis occurred without a marked block in cell cycle progression at the time point evaluated. This pattern suggests that, at the concentration and time point tested, MfEA may engage the apoptotic machinery without a detectable prior checkpoint arrest.

Under the conditions evaluated, the apoptotic response elicited by MfEA in SCC-9 cells occurred without detectable H_2_O_2_ accumulation and without a preceding cell cycle arrest, in line with the activity described for the phenolic acids, hydrolyzable tannins, and triterpenoids tentatively identified in the fraction. Given its pronounced and selective cytotoxic activity, the safety profile of MfEA was next characterized in vitro and in vivo.

### 2.4. MfEA Exhibits Low Hemolytic Activity and a Dose-Dependent Toxicity Profile In Vivo

To characterize the safety profile of MfEA, two complementary assays were performed. Hemolytic activity was first assessed in human erythrocytes to determine whether its cytotoxic effects could be associated with nonspecific membrane disruption or surfactant-like properties. An in vivo acute toxicity assay was then conducted in C57BL/6 mice to investigate its systemic effects and overall tolerability.

**Figure 3:**
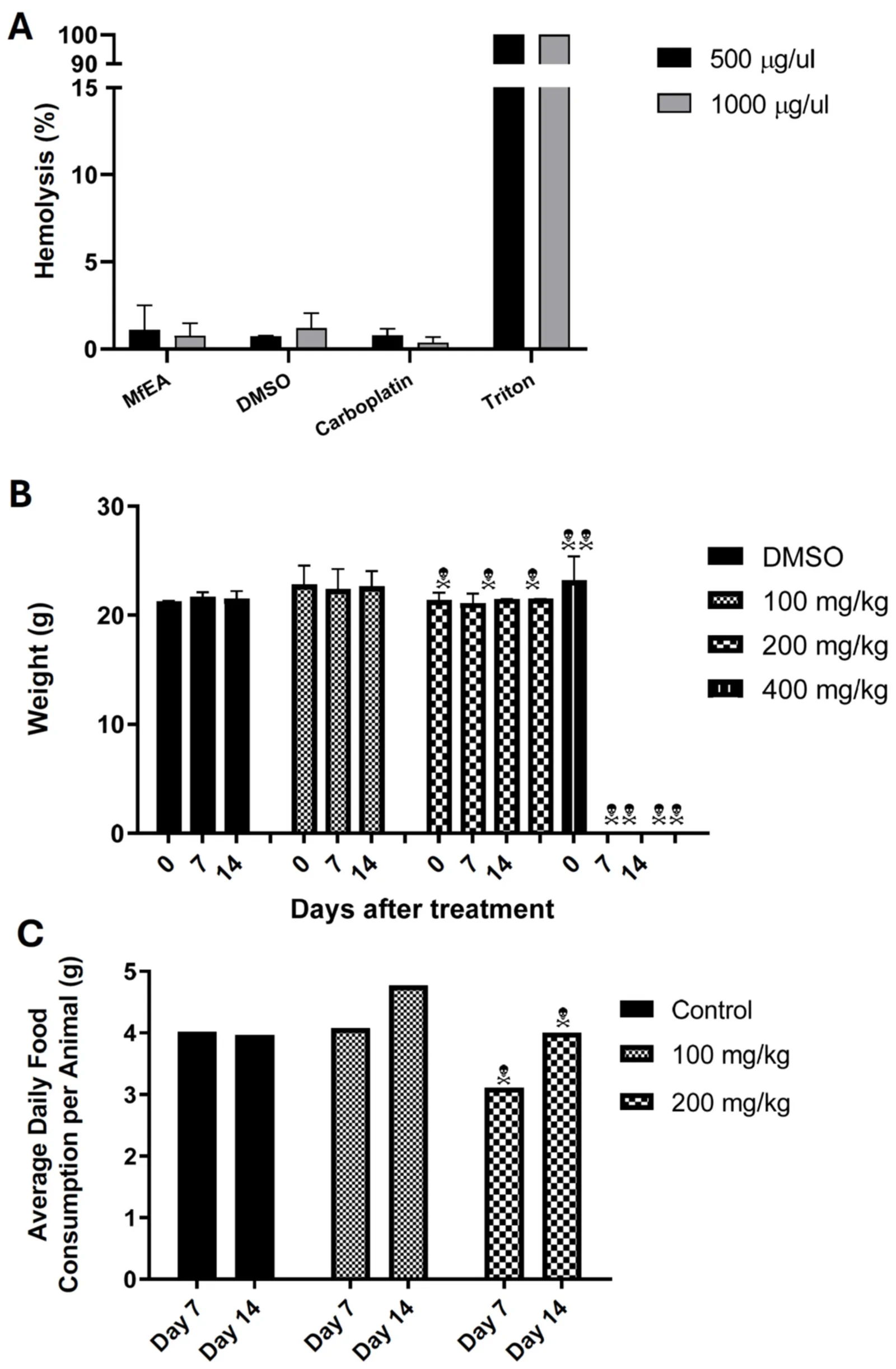
Hemolytic activity and in vivo acute toxicity profile of MfEA. (**A**) Hemolytic activity in human erythrocytes, quantified by spectrophotometric measurement at 540 nm. DMSO was used as the negative control, Triton X-100 as the positive control (100% hemolysis), and carboplatin as a reference chemotherapeutic agent. (**B**) Body weight variation in mice over 14 days following treatment with MfEA or vehicle control (DMSO). (**C**) Average daily food intake of mice during the 14-day treatment period. Mortality events are indicated by symbols: a single skull symbol denotes the death of two of the four animals in the 200 mg/kg group within 48 h after treatment, and a double skull symbol denotes the death of all three animals in the 400 mg/kg group within 48 h.

MfEA exhibited very low hemolytic activity (<3%) at 500 and 1000 µg/mL, comparable to that observed for carboplatin and the DMSO vehicle (Figure 3A). This result indicates minimal erythrocyte membrane disruption and suggests that MfEA-induced cytotoxicity is not mediated by nonspecific surfactant-like activity.

The low hemolytic activity of MfEA is consistent with the behavior described for the phenolic compounds putatively annotated in this fraction. Ellagic acid (**4**) has been shown to protect the human erythrocyte membrane against oxidative injury, reducing lysis and preserving membrane-bound enzyme activity [90], and extracts combining gallic acid, ellagic acid, and hydrolyzable tannins likewise protect erythrocytes from oxidative hemolysis [91]. Similarly, polyphenol-rich extracts containing hydrolyzable tannins stabilize erythrocyte membranes under oxidative and hypotonic stress [92]. The negligible hemolysis observed for MfEA at concentrations up to 23-fold higher than its IC_50_ in SCC-9 cells is therefore in line with the behavior described for this class of metabolites.

Acute toxicity studies are an essential step in preclinical evaluation, providing information on safe, toxic, and potentially lethal doses, as well as insights into possible mechanisms of toxicity [93]. Given the low hemolytic activity (<3%) and the previously documented selectivity of MfEA toward cancer cell lines, this fraction was considered suitable for in vivo toxicity assessment.

MfEA was administered intraperitoneally to C57BL/6 mice at doses of 100, 200, and 400 mg/kg, with a vehicle control group receiving PBS containing 3% DMSO. Animals were monitored for 14 days following treatment, and each subsequent dose was administered only after completion of the observation period of the preceding group.

At 100 mg/kg, no mortality or signs of morbidity were observed. Animals maintained normal behavior throughout the experimental period, and necropsy detected no macroscopic alterations. Body weight and food intake remained comparable to those of the control group.

At 200 mg/kg, transient lethargy was observed shortly after administration. One animal died within 48 h, while the remaining animals recovered normal behavior and survived until the end of the observation period. Necropsy revealed localized alterations in the gastrointestinal tract and gallbladder in two animals. An additional animal added to the 200 mg/kg group (*n* = 4 in total) presented the same initial signs of toxicity: lethargy, reduced mobility, local bleeding, and pain-related behaviors. This animal died within 24 h. At 400 mg/kg, all animals exhibited severe lethargy shortly after treatment and died within 48 h, precluding reliable macroscopic evaluation.

No relevant changes in body weight were observed across groups compared to controls (Figure 3B). At 100 mg/kg, food intake remained comparable to the control group on day 7 and was slightly higher on day 14. In contrast, at 200 mg/kg, food intake was reduced on day 7 but returned to control levels by day 14, suggesting a transient effect on feeding behavior (Figure 3C). Deceased animals were excluded from these analyses. Detailed data can be accessed at Supplementary Table S1.

Based on these findings, the approximate lethal dose of MfEA by the intraperitoneal route (LD_50_) is estimated to lie close to 200 mg/kg. The 100 mg/kg dose was well tolerated under the conditions tested, with no mortality, morbidity, or macroscopic alteration detected at this dose in vivo. Although further studies are needed to elucidate the molecular basis of the toxic effects observed at higher doses, MfEA combined pronounced in vitro cytotoxicity with tolerability at 100 mg/kg in vivo, justifying continued investigation as an anticancer candidate.

The intraperitoneal LD_50_ estimated for MfEA is comparable to that reported for other polyphenol-rich extracts of similar chemical composition. A standardized pomegranate extract, rich in ellagitannins, ellagic acid, and gallic acid, showed an intraperitoneal LD_50_ of 187 mg/kg in mice, whereas its oral LD_50_ exceeded 5 g/kg [94]. This marked difference between administration routes indicates that the toxicity observed for this class of extracts is strongly route-dependent, which is consistent with the low toxicity described for isolated constituents such as gallic acid, whose oral LD_50_ in mice exceeds 2000 mg/kg [95]. The localized gastrointestinal alterations observed at 200 mg/kg are compatible with direct exposure of the peritoneal cavity to the administered fraction.

Together, these results indicate that MfEA shows no evidence of nonspecific membrane damage at the cellular level and is tolerated in vivo at doses within the range reported for structurally related polyphenol-rich extracts.

## 3. Materials and Methods

### 3.1. Plant Extract Preparation and Analysis

#### 3.1.1. Plant Material

Plant materials were collected in the Restinga de Jurubatiba National Park, located in Carapebus, Rio de Janeiro State, Brazil. The Brazilian environmental and genetic heritage authorities authorized collection and research activities involving plant materials under SisBio/ICMBio permit No. 13659-22 and SisGen registrations A491A56 and A314288. The botanist Marcelo Guerra Santos identified the collected specimens and deposited voucher specimens in the Herbarium of the Faculty of Teaching at the State University of Rio de Janeiro (FFP/UERJ). Table 6 provides information on scientific names, collection coordinates, voucher numbers, plant parts analyzed, and quantities collected.

#### 3.1.2. Extraction and Liquid Partition

Leaves and stems from three individuals of each species were pooled before drying and extraction, and each extract and fraction was prepared as a single batch that was used for all subsequent chemical and biological analyses. The plant materials were dried in a forced-air circulation oven (model LB 82-1152, Labtec, Ribeirão Preto, SP, Brazil) at 35 ± 2 °C for 24 to 48 h to reduce moisture content and stabilize them. Then, the dried leaves or stems were ground using a hammer mill and extracted by maceration with 96% (*v*/*v*) ethanol at room temperature (25 °C). Four successive extraction cycles were performed, each lasting seven days with solvent renewal. The extracts were filtered and concentrated under reduced pressure using a rotary evaporator (Model 802, Fisatom, São Paulo, SP, Brazil). The concentrated extract was subsequently lyophilized to dryness to obtain the crude ethanolic extract.

The biologically active crude ethanolic extracts from leaves or stems were subsequently subjected to liquid–liquid partitioning using organic solvents of increasing polarity (hexane, dichloromethane, and ethyl acetate). The resulting fractions were concentrated under reduced pressure, freeze-dried, and stored at −20 °C until further use.

#### 3.1.3. Chemical Characterization by UPLC-DAD-QTOF-ESI-HRMS/MS

The most promising fraction was analyzed by ultra-performance liquid chromatography coupled with diode-array detection and high-resolution time-of-flight electrospray ionization mass spectrometry (UPLC-DAD-QTOF-ESI-HRMS). Analyses were performed on a Shimadzu (Shimadzu Corporation, Kyoto, Japan) UPLC-DAD-QTOF-ESI-HRMS system equipped with a Kinetex C18 column (150 mm × 4.6 mm, 2.6 μm particle size; Phenomenex, Torrance, CA, USA). Chromatographic separation was achieved using a binary mobile phase composed of solvent A (deionized water containing 0.1% formic acid) and solvent B (HPLC-grade acetonitrile, Tedia Company, Fairfield, OH, USA). The elution program was as follows: 0–42 min, 0–100% B; 42–46 min, 100% B; and 46–54 min, 100–3% B, at a flow rate of 0.4 mL/min. Mass spectrometric detection was performed on a Bruker Compact Q-TOF high-resolution mass spectrometer over an *m*/*z* range of 50–1200 (Bruker Daltonics, Bremen, Germany). Electrospray ionization (ESI) was performed in both positive and negative ion modes. Sodium formate was used for mass calibration. The ESI source parameters were set as follows: nebulizer gas (N_2_), 5.5 bar; dry gas (N_2_), 12 L/min at 220 °C; and capillary voltage, 4.5 kV. Data acquisition and processing were performed using Bruker Compass DataAnalysis software version 4.4.

### 3.2. Biological Assays

#### 3.2.1. Cells and Reagents

The human SCC-4, SCC-9, and SCC-25 cells, derived from human oral squamous cell carcinoma, were obtained from the American Type Culture Collection (ATCC, Manassas, VA, USA) (CRL-1624; CRL-1629, and CRL-1628, respectively) and maintained in 1:1 DMEM/F12 medium (Dulbecco’s modified Eagle’s medium; Gibco, Thermo Fisher Scientific, Waltham, MA, USA) supplemented with 10% (*v*/*v*) FBS (fetal bovine serum; Invitrogen, Thermo Fisher Scientific, Waltham, MA, USA) and 400 ng/mL of hydrocortisone (Sigma-Aldrich Co., St. Louis, MO, USA). Also obtained from ATCC, primary normal human gingival fibroblast (PCS-201-018), HeLa (CRM-CCL-2), HepG2 (HB-8065), HCT 116 (CCL-247), 4T1 (CRL-2539) and B16-F10 (CRL-6475) were maintained in DMEM supplemented with 10% (*v*/*v*) FBS and were used at a maximum of 6 passages. Cells were cultured in a humidified incubator at 37 °C with 5% CO_2_. For all biological experiments, extracts and fractions were solubilized in 100% DMSO (Sigma-Aldrich Co., St. Louis, MO, USA) to a final concentration of 25 mg/mL. For test concentrations above 300 µg/mL, a 100 mg/mL stock solution was used instead, so as to reduce the final DMSO concentration in the well. Carboplatin in water (Fauldcarbo^®^; Libbs Farmacêutica, São Paulo, SP, Brazil) was used as a standard anticancer compound.

#### 3.2.2. Cell Viability Assay (Cytotoxicity)

The SCC cell lines, HeLa, HepG2, HCT 116, 4T1, B16-F10, and primary human fibroblasts (viability) were evaluated using the MTT assay, as previously described by our group [96]. Cells were seeded in triplicate in 96-well plates (5 × 10^3^ cells/well) and grown for 24 h. Then, medium was removed, fresh medium was added, and the cells were returned to the incubator in the presence of different extracts and chemical fractions at different concentrations. DMSO at the same concentrations as the treatments served as the 100% cell viability control. After 48 h, cells were incubated with 0.5 mg/mL MTT reagent (3-(4,5-dimethyl-2-thiazolyl)-2,5-diphenyl-2-H-tetrazolium bromide) (Sigma-Aldrich Co., St. Louis, MO, USA) for 3.5 h. Thereafter, the formazan crystals were dissolved in MTT solvent solution (DMSO/methanol 1:1 *v*/*v*), and the absorbance at 560 nm was evaluated using an EPOCH Microplate Spectrophotometer (BioTek Instruments, Winooski, VT, USA) with the background absorbance at 670 nm subtracted. Each of the 17 stems and leaves crude ethanolic extracts (stem and leaves) was tested at six, eight or eleven different concentrations varying from 1.02 μg/mL to 562.5 μg/mL in cancer cell lines and 31.24 μg/mL to 2000 μg/mL in normal human gingival fibroblast. The positive control (carboplatin) was tested at six different concentrations varying from 11.66 μg/mL to 500 μg/mL.

#### 3.2.3. Statistical Analysis and IC_50_ Calculation

IC_50_ values for the MTT assay were obtained by nonlinear regression of a log(inhibitor) vs. response curve, fitted by least squares in GraphPad Prism 8.0.2 (GraphPad Software, San Diego, CA, USA), from three independent experiments. Data are presented as the IC_50_ with its 95% confidence interval (95% CI) and the coefficient of determination (R^2^), the interval being obtained by back-transforming the logIC_50_ estimate and its standard error (logIC_50_ ± 1.96 × SE) returned by the regression. The selectivity index (SI) was calculated as IC_50_ in normal gingival fibroblasts/IC_50_ in each cancer cell line, and the average was calculated when indicated. The caspase-3/7, cell cycle and sub-G0/G1, Annexin V/7-AAD, ROS and time-lapse videomicroscopy assays were each performed in three independent experiments, and the panels shown are representative of those experiments.

#### 3.2.4. Detection of Caspase-3/7 Activity

Effector caspase-3/7 activation following treatment with the ethyl acetate fraction of *M. floribunda* stem (MfEA) was evaluated using CellEvent™ Caspase-3/7 Red Detection Reagent (Thermo Fisher Scientific, Waltham, MA, USA), according to the manufacturer’s instructions. SCC-9 cells (5 × 10^4^) were seeded in 24-well plates and incubated for 24 h prior to treatment with MfEA at 2 × IC_50_ (85.8 µg/mL) or DMSO (negative control) for 48 h. Cells were then stained according to the manufacturer’s protocol. Fluorescence was measured using a Guava^®^ Muse Cell Analyzer flow cytometer (Cytek Biosciences, Fremont, CA, USA). Data were analyzed using Flowing Software v2.5.1 (Turku Biosciences, Turku, Finland).

#### 3.2.5. Cell Cycle and Sub-G_0_/G_1_ Analysis

To evaluate the effects of MfEA on cell cycle distribution and DNA fragmentation (sub-G_0_/G_1_), SCC-9 cells (1.5 × 10^5^ cells/well) were seeded in 6-well plates and treated at 2 × IC_50_ or DMSO (negative control) for 48 h. Following treatment, cells were trypsinized and stained with a propidium iodide buffer (pH 7.6) composed of 3.4 mM Tris–HCl, 10 mM NaCl, 0.1% (*v*/*v*) NP-40, 700 U/L RNase, and 0.075 mM propidium iodide (all from Sigma-Aldrich Co., St. Louis, MO, USA), as previously described [97]. DNA content was analyzed by acquiring 10,000 events using a Guava^®^ Muse Cell Analyzer flow cytometer (Cytek Biosciences, Fremont, CA, USA). Data were analyzed using Flowing Software v2.5.1 (Turku Biosciences, Turku, Finland).

#### 3.2.6. Phosphatidylserine Exposure Detection

Phosphatidylserine externalization was evaluated by flow cytometry using the Annexin V Apoptosis Detection Kit with 7-AAD (Thermo Fisher Scientific, Waltham, MA, USA), according to the manufacturer’s instructions. Briefly, SCC-9 cells (1.5 × 10^5^ cells/well) were seeded in 6-well plates and treated with MfEA at 2 × IC_50_ (85.8 µg/mL) or DMSO (negative control) for 48 h. Cells were then trypsinized, washed twice with cold PBS, and resuspended in Annexin V binding buffer. Aliquots were incubated with FITC-conjugated Annexin V and 7-AAD for 15 min at room temperature in the dark. A minimum of 10,000 events was acquired on a Guava^®^ Muse Cell Analyzer flow cytometer (Cytek Biosciences, Fremont, CA, USA), and data were analyzed with Flowing Software v2.5.1 (Turku Biosciences, Turku, Finland).

#### 3.2.7. Hydrogen Peroxide (H_2_O_2_) Quantification

For the H_2_O_2_ quantification assay, the ROS-Glo H_2_O_2_ Assay Kit (Promega, Madison, WI, USA) was used. In this assay, 1 × 10^4^ cells/well were plated in a 96-well plate. The cells were incubated for 24 h, after which the medium was removed, and the cells were treated with 2 × IC_50_ (85.8 μg/mL) of MfEA, DMSO (negative control), or 20 μM of menadione (Sigma-Aldrich Co., St. Louis, MO, USA; positive control). A cell-free well was used as a control. The treatment time was 12, 24, and 36 h. Two hours before the end of the treatment time, H_2_O_2_ substrate was added. After the end of the treatment time, the detection solution containing D-cysteine, signal amplifier, and luciferin was added, following the kit protocol. The supernatant was collected, and light emission was quantified using a TD-20/20 luminometer (Turner Designs, Sunnyvale, CA, USA).

#### 3.2.8. Time-Lapse Videomicroscopy

SCC-9 cells, plated 24 h before the experiment, were treated and transferred to a culture chamber adapted to a Leica DMI1 microscope (Leica Microsystems, Wetzlar, Germany) under controlled conditions of CO_2_ and temperature (5% and 37 °C, respectively). For 72 h, phase contrast images from the same field were captured every 1 min and 36 s. Two experimental conditions were performed: control (DMSO) and treatment with MfEA at 2 × IC_50_ (85.8 μg/mL). Images of each experimental condition were compiled into videos using the Framelapse Pro application (version 4.0; Neximo Labs, Noida, Uttar Pradesh, India).

#### 3.2.9. Hemolysis Assay

Human blood was used as approved by the Research Ethics Committee of the Fluminense Federal University–Nova Friburgo, RJ (CAAE: 77584924.0.0000.5626). Erythrocytes were collected, centrifuged at 1500 rpm for 15 min, washed with PBS (phosphate-buffered saline) and 10 mM glucose, and counted in a Countess II cell counter (Thermo Fisher Scientific, Waltham, MA, USA). Erythrocytes were then plated in 96-well plates at a concentration of 4 × 10^8^/well (in PBS with 10 mM glucose, 100 μL final volume) in triplicate, and the MfEA and carboplatin were added at a final concentration of 500 μg/mL and 1000 μg/mL. DMSO served as the negative control, and 0.1% Triton X-100 as the positive control. Absorbance was read on an EPOCH Microplate Spectrophotometer (BioTek Instruments, Winooski, VT, USA) at 540 nm, and statistical analysis was performed with the GraphPad Prism 8.0.2 software (GraphPad Software, San Diego, CA, USA).

#### 3.2.10. In Vivo Acute Toxicity Study

The acute toxicity study of MfEA was conducted in twelve-week-old female C57BL/6 mice via the intraperitoneal route and was approved by the University Animal Ethics Council (CEUA #2699110419). All experiments were performed in accordance with Brazilian guidelines and regulations. The group assigned to the 200 mg/kg dose included *n* = 4 animals, while the others had *n* = 3 animals each and received a single intraperitoneal injection (Day 0) of each compound dissolved in PBS with 3% DMSO. Control animals received only 3% of DMSO in PBS. The first dose of the compound was 100 mg/kg. Subsequent dose levels (200 mg/kg and 400 mg/kg) were determined based on the results of the previous dose. Animals were monitored twice daily for mortality and morbidity for 14 days, after which all animals were euthanized by cervical dislocation and subjected to gross necropsy. Animal body weight and average food consumption were measured every 7 days. As morbidity indicators, the following signs were assessed twice daily: tremors; convulsions; salivation; diarrhea; lethargy; coma; signs of pain; increased back arching and mobility defect. The necropsy included an examination of the external features of the carcass; external body orifices; the abdominal and thoracic cavities; and the liver, spleen, kidneys, heart, and lungs.

#### 3.2.11. Use of Artificial Intelligence Tools

Artificial intelligence (AI) assistance was employed to improve the clarity, coherence, and overall fluency of the manuscript. Specifically, the language model Claude Opus 5 (Anthropic, San Francisco, CA, USA) was used for text editing, grammatical refinement, and stylistic enhancement. The AI tool was also utilized for general writing support tasks consistent with accepted scientific publishing practices, including improving readability and organization of the text. All scientific content, interpretations, and conclusions were critically reviewed and validated by the authors, who take full responsibility for the integrity and accuracy of the work.

## 4. Conclusions

In the present work, seventeen extracts derived from ten plant species native to Restinga ecosystems of the Brazilian Atlantic Forest were screened against the OSCC cell line SCC-9, and the ethyl acetate fraction of *Myrciaria floribunda* stem (MfEA) was identified as the most potent and selective cytotoxic fraction. Chemical profiling of MfEA putatively annotated phenolic acids, hydrolyzable tannins, and pentacyclic triterpenoids, classes consistently associated with antitumor activity. Across a panel of eight tumor cell lines, MfEA was, on average, more potent and more selective than carboplatin under the in vitro conditions tested; in SCC-9 cells, it induced morphological and biochemical hallmarks of apoptosis, including caspase-3/7 activation, sub-G_0_/G_1_ accumulation, and phosphatidylserine externalization, without detectable H_2_O_2_ accumulation.

MfEA displayed negligible hemolytic activity and was well tolerated in vivo at 100 mg/kg, with an estimated intraperitoneal LD_50_ close to 200 mg/kg. Together, these findings identify MfEA as a promising lead for the development of selective anticancer agents against OSCC. The evaluation of its antitumor efficacy in xenograft models, together with the isolation of the active constituents and the characterization of the molecular pathways underlying its apoptotic effect, represents the natural continuation of this work.

## Figures and Tables

**Table 1:** Extraction yields (%, *w*/*w*) and masses (g) of crude ethanolic extracts and liquid–liquid partition fractions (g) obtained from the Restinga de Jurubatiba National Park plant species.

Plant	Ethanolic Extracts (g)		Yield (%, *w*/*w*)
*Myrciaria floribunda*	Leaves	1087.0	21.74
	Stems	319.3	8.24
*Myrcia amazonica*	Leaves	249.1	17.06
	Stems	86.4	10.67
*Myrsine parvifolia*	Leaves	690	25.56
	Stems	138.4	5.77
*Myrcia ovata*	Leaves	311.5	20.00
	Stems	69.4	7.80
*Pilocarpus spicatus*	Leaves	51.0	7.04
*Eugenia sulcata*	Leaves	340	21.25
*Eugenia pruniformis*	Leaves	143.7	11.98
	Stems	37.5	5.47
*Ocotea indecora*	Leaves	223.6	20.33
	Stems	34.3	5.19
*Ocotea notata*	Leaves	202	18.30
	Stems	103.4	12.02
*Ocotea pulchella*	Leaves	81.5	5.68
	**Fraction extracts (g)**		**Yield (%, *w*/*w*)**
*Myrciaria floribunda*	Leaves		
Hexane		212.6	4.25
Dichloromethane		151.5	3.03
Ethyl acetate		181.5	3.63
*Myrciaria floribunda*	Stems		
Hexane		11.5	3.83
Dichloromethane		16.4	5.47
Ethyl acetate		9.8	3.27

**Table 2:** Cytotoxicity of seventeen different ethanolic extracts in SCC-9 cells. The half-maximal inhibitory concentration (IC_50_), 95% confidence interval (95% CI), and coefficient of determination (R^2^) were calculated using non-linear regression in GraphPad Prism 8. Data were collected from at least three independent experiments. IC_50_ in µg/mL. R^2^ = coefficient of determination. 95% CI = 95% confidence interval of the IC50. N/D = not determined.

Extracts	SCC-9			Gingival Fibroblasts			Selectivity Index
	IC_50_	R^2^	95% CI	IC_50_	R^2^	95% CI	
*Myrciaria floribunda* Leaves	78.72	0.75	67.4–98	562.5	0.73	491–644	7.14
*Myrciaria floribunda* Stem	56.90	0.77	47.6–68.3	387.6	0.82	313–502	6.80
*Myrcia amazonica* Leaves	N/D	N/D	N/D	-	-	-	-
*Myrcia amazonica* Stem	N/D	N/D	N/D	-	-	-	-
*Myrsine parvifolia* Leaves	N/D	N/D	N/D	-	-	-	-
*Myrsine parvifolia* Stem	N/D	N/D	N/D	-	-	-	-
*Myrcia ovata* Leaves	169.0	0.79	141–202	-	-	-	-
*Myrcia ovata* Stem	N/D	N/D	N/D	-	-	-	-
*Pilocarpus spicatus* Leaves	153.8	0.72	102–231	-	-	-	-
*Eugenia sulcata* Leaves	129.8	0.80	113–149	214.7	0.77	171–269	1.65
*Eugenia pruniformis* Leaves	141.9	0.78	124–162	228.9	0.78	183–287	1.61
*Eugenia pruniformis* Stem	106.4	0.80	88.8–127	341.4	0.63	238–490	3.21
*Ocotea indecora* Leaves	120.7	0.62	96.3–151	177	0.75	135–232	1.47
*Ocotea indecora* Stem	108.8	0.71	90.8–130	279.6	0.75	213–367	2.57
*Ocotea notata* Leaves	N/D	N/D	N/D	-	-	-	-
*Ocotea notata* Stem	N/D	N/D	N/D	-	-	-	-
*Ocotea pulchella* Leaves	N/D	N/D	N/D	-	-	-	-
Carboplatin	97.70	0.92	89.3–107	131.9	0.69	87.9–198	1.35

**Table 3:** Cytotoxicity and selectivity of fractions derived from *Myrciaria floribunda* leaf and stem ethanolic extracts in SCC cell lines. The half-maximal inhibitory concentration (IC_50_), its 95% confidence interval (95% CI), and the coefficient of determination (R^2^) were calculated by non-linear regression in GraphPad Prism 8. Data were collected from at least three independent experiments. IC_50_ in µg/mL. 95% CI = 95% confidence interval of the IC_50_. R^2^ = coefficient of determination. SI = selectivity index (IC_50_ in fibroblasts/IC_50_ in the tumor cell line); values above 2 are shown in bold. N/D = not determined; – = not applicable.

Fraction	Fibroblast		SCC-9			SCC-4			SCC-25		
	IC_50_ (95% CI)	R^2^	IC_50_ (95% CI)	R^2^	SI	IC_50_ (95% CI)	R^2^	SI	IC_50_ (95% CI)	R^2^	SI
***Myrciaria floribunda* leaves**											
Hexane	68.4 (54.6–85.7)	0.68	19.6 (15.0–25.7)	0.90	3.49	38.1 (29.1–49.9)	0.81	1.79	36.3 (25.3–52.1)	0.68	1.88
Dichloromethane	62.1 (49.6–77.8)	0.73	38.8 (31.0–48.6)	0.90	1.60	N/D	–	**–**	N/D	–	**–**
Ethyl acetate	N/D	–	N/D	–	**–**	N/D	–	**–**	N/D	–	**–**
***Myrciaria floribunda* stem**											
Hexane	52.5 (45.9–60.1)	0.87	19.3 (15.4–24.2)	0.94	2.72	60.4 (50.4–72.3)	0.89	0.87	57.1 (54.6–59.7)	0.58	0.92
Dichloromethane	65.1 (56.9–74.5)	0.90	14.4 (13.8–15.1)	0.70	4.52	23.1 (18.4–28.9)	0.87	2.81	21.4 (14.3–32.1)	0.70	3.04
**Ethyl acetate (MfEA)**	568.0 (414–779)	0.49	42.9 (32.7–56.2)	0.84	13.24	71.3 (56.9–89.3)	0.89	7.97	60.5 (46.1–79.3)	0.82	9.39
Carboplatin	131.9 (87.3–198)	0.69	97.70 (89.3–107)	0.92	1.35	59.9 (47.8–75.1)	0.90	2.20	67.1 (46.8–96.3)	0.71	1.97

**Table 5:** Cytotoxicity and selectivity of *Myrciaria floribunda* ethyl acetate fraction (MfEA) across eight different cancer cell lines. The half-maximal inhibitory concentration (IC_50_), 95% confidence interval (95% CI), and coefficient of determination (R^2^) were calculated using non-linear regression in GraphPad Prism 8. Data were collected from at least three independent experiments. IC_50_ in µg/mL. R^2^ = coefficient of determination. 95% CI = 95% confidence interval of the IC_50_. SI = selectivity index.

Cell Line	*Myrciaria floribunda* Stem Ethyl Acetate Fraction (MfEA)				Carboplatin			
	IC_50_	R^2^	95% CI	SI	IC_50_	R^2^	95% CI	SI
Fibroblast	568.0	0.49	414–779	-	131.9	0.69	87.3–198	-
SCC-9	42.9	0.84	32.7–56.2	13.2	97.70	0.92	89.3–107	1.35
SCC-4	71.3	0.89	56.9–89.3	7.97	59.9	0.90	47.8–75.1	2.18
SCC-25	60.5	0.82	46.1–79.3	9.39	67.1	0.71	46.8–96.3	1.95
B16-F10	92.7	0.90	67.6–127	6.14	117.4	0.89	93.7–147	1.12
HCT 116	61.3	0.79	39–96.3	9.28	191.8	0.70	128–288	0.69
HeLa	29.8	0.88	21.7–40.9	19.1	48.7	0.94	38.9–61	2.70
HepG2	45.2	0.88	31.5–64.9	12.6	13.3	0.98	11.6–15.2	9.92
4T1	9.4	0.87	6.55–13.5	60.5	37.4	0.84	24.9–56.1	3.53

**Table 6:** Scientific names, botanical families, vernacular names, collection site coordinates, voucher specimen numbers, plant parts, and amounts of dried plant material of the Restinga species investigated.

Species	Family	Vernacular Name	Collection Coordinates	Voucher	Plant Part	Dried Material (g)
*Myrciaria floribunda*	Myrtaceae	“cambuí”, “camboim”, “camboim amarelo”	22°14′074″ S, 41°35′727″ W 22°13′7.20″ S, 41°35′8.50″ W	RFFP 13.789	Leaves	5000
			22°13′065″ S, 41°35′163″ W		Stems	3876
*Myrcia amazonica*	Myrtaceae	“camboim-mirim”	22°13′0740″ S, 41°35′1661″ W 22°13′0460″ S, 41°35′1530″ W	RFFP 19.802	Leaves	1460
			22°13′7.30″ S, 41°35′8.70″ W		Stems	810
*Myrcia ovata*	Myrtaceae	“laranjinha-do-mato”	22°12′701″ S, 41°35′172″ W	RFFP 14250	Leaves	1560
			22°13′057″ S, 41°39′197″ W 22°12′701″ S, 41°39′167″ W		Stems	890
*Eugenia sulcata*	Myrtaceae	“pitanguinha”, “murtinha”	22°12′57.7″ S, 41°34′58.5″ W 22°12′670″ S, 41°35′25″ W 22°13′0.52″ S, 41°34′995″ W	RFFP 13.788	Leaves	1600
*Eugenia pruniformis*	Myrtaceae	“azeitoninha-da-praia”	22°12′717″ S, 41°35′289″ W	M.G. Santos 2206	Leaves	1200
			22°13′9.40″ S, 41°35′9.50″ W 22°12′42″ S, 41°35′15.9″ W		Stems	685.7
*Myrsine parvifolia*	Primulaceae	“capororoca”, “capororoquinha”	22°14′46″ S, 41°34′56″ W 22°13′063″ S, 41°35′166″ W	RFFP 15316	Leaves	2700
			22°13′04.7″ S, 41°35′24.1″ W		Stems	2400
*Pilocarpus spicatus*	Rutaceae	“jaborandi-da-restinga”	22°14′00.2″ S, 41°35′43.8″ W 22°13′59.0″ S, 41°35′45.0″ W 22°13′05.7″ S, 41°35′17.7″ W	RFFP 17.339	Leaves	724
*Ocotea indecora*	Lauraceae	“canela sassafrás”	22°12′41.2″ S, 41°35′17.7″ W 22°12′68.6″ S, 41°35′29.40″ W	RFFP 16.873	Leaves	1100
			22°12′41.1″ S, 41°35′17.1″ W		Stems	660.4
*Ocotea notata*	Lauraceae	“canela branca”	22°14′121″ S, 41°35′807″ W 22°13′0.10″ S, 41°35′4.00″ W	M.G. Santos 2296	Leaves	1104
			22°13′2.10″ S, 41°35′9.00″ W		Stems	860
*Ocotea pulchella*	Lauraceae	“canelinha”, “canela-preta”	22°12′58.90″ S, 41°35.14.2″ W 22°13′14.4″ S, 41°34′59.8″ W 22°13′18.90″ S, 41°35′2.60″ W	RFFP 16451	Leaves	1434

RFFP = Herbarium of the Faculdade de Formação de Professores, Universidade do Estado do Rio de Janeiro.

## Data Availability

All data supporting the findings of this study, including individual dose–response curves, flow-cytometry files and individual-animal data, are available from the corresponding author upon reasonable request.

## Supplementary Materials

The following supporting information can be downloaded at: https://www.mdpi.com/article/10.3390/molecules31213976/s1, Supplementary Table S1: Outcomes observed in control and MfEA-treated groups, including variations in body weight and food consumption, signs of morbidity, mortality and macroscopic alterations in the necropsied organs.
