# Supplementary material for: Bioprospecting of Brazilian Restinga Plants Reveals a Myrciaria floribunda Fraction with Apoptotic Activity Against Oral Carcinoma Cells
Source: Molecules. 2026 Nov 4;31(21):3976. doi: 10.3390/molecules31213976 (PMC13764731; doi:10.3390/molecules31213976)
Supplement: Supplementary file 1 [file molecules-31-03976-s001.zip › molecules-4660474-supplementary.pdf]

molecules

MDPI

Supplementary Materials

Bioprospecting of Brazilian Restinga plants reveals a *Myrciaria floribunda* fraction with apoptotic activity against oral carcinoma cells

Julio Cesar Thurler-Júnior [1,2,†], Francisco Paiva Machado [2,3,†], Luiza Soares Batista [1], Diogo Folly Gomes Andrade [3], Gabriel Ouverney [1,4], Marcelo Guerra Santos [5], Anamel Blaudt Meira [1], Guilherme Freimann Wermelinger [1], Leandro Rocha [3,*] and Bruno Kaufmann Robbs [1,*]

1 Laboratório de Rastreamento Biológico de Novas Drogas Contra o Câncer (CancerBioScreen), Departamento de Ciências Básicas, Instituto de Saúde de Nova Friburgo (ISNF), Universidade Federal Fluminense (UFF), Nova Friburgo, RJ, Brazil
2 Programa de Pós-Graduação em Ciências Aplicadas a Produtos para a Saúde, Faculdade de Farmácia, Universidade Federal Fluminense, Niterói, RJ, Brazil;
3 Laboratório de Tecnologia de Produtos Naturais (LTPN), Faculdade de Farmácia, Universidade Federal Fluminense, Niterói, RJ, Brazil;
4 Programa de Pós-Graduação em Ciências Morfológicas, Instituto de Ciências Biomédicas, Universidade Federal do Rio de Janeiro, Rio de Janeiro, RJ, Brazil;
5 Departamento de Ciências da Faculdade de Formação de Professores (FFP), Universidade do Estado do Rio de Janeiro, Rio de Janeiro, RJ, Brazil.
* Correspondence: leandromr@id.uff.br (L.M.R.); brunokr@id.uff.br (B.K.R.); Tel.: +55-21-99619-8584 (L.M.R.); +55-21-99772-1259 (B.K.R.)
† These authors contributed equally to this work.

Table S1. Outcomes observed in control and MfEA-treated groups, including variations in body weight and food consumption, signs of morbidity, mortality and macroscopic alterations in the necropsied organs. Each experimental group consisted of three C57BL/6 mice (*n* = 3), except the 200 mg/kg group, which had *n* = 4. Animals were monitored for 14 days, after which surviving animals were euthanized and subjected to necropsy. Body weight and food consumption curves are shown in Figure 2B,C of the main text.

Treatment	Dose (mg/kg)	Change in body weight	Change in food consumption	Morbidity	Mortality	Gross necropsy
**Control**	0 (*n* = 3)	Day 0: 21.27 ± 0.05 Day 14: 21.50 ± 0.71 (+1.1%)	Day 7: 4.02 Day 14: 3.97	Absent	Absent	Absent
**MfEA**	100 (*n* = 3)	Day 0: 22.81 ± 1.75 Day 14: 22.66 ± 1.40 (−0.7%)	Day 7: 4.08 (+1.5%) Day 14: 4.77 (+20.2%)	Absent	Absent	Absent
	200 (*n* = 4)	Day 0: 21.39 ± 0.66 Day 14: 21.48 ± 0.03 (+0.4%)	Day 7: 3.12 (−22.4%) Day 14: 4.00 (+0.8%)	Transient lethargy, reduced mobility and pain behavior.	2/4 (within 48 h)	Intestine and gallbladder alterations
	400 (*n* = 3)	Not evaluated	Not evaluated	Severe lethargy, reduced mobility and pain behavior.	3/3 (within 48 h)	Not evaluated

MfEA = ethyl acetate fraction of *Myrciaria floribunda* stems. Doses were administered as a single intraperitoneal injection on day 0 and animals were observed for 14 days. Mortality is expressed as the number of animals found dead over the number of animals in the group. At 400 mg/kg, body weight, food consumption and gross necropsy could not be evaluated because all animals died within 48 h of administration. Body weight is expressed as mean ± SD (g); the percentage in parentheses is the change from day 0 within the same group. Food consumption is expressed as mean daily intake (g); the percentage in parentheses is the difference relative to the control group at the same time point.
